# Can lingual spurs alter the oral health-related quality of life during anterior open bite interceptive treatment? A systematic review

**DOI:** 10.1590/2177-6709.28.1.e2321298.oar

**Published:** 2023-04-14

**Authors:** Larissa Barbosa MODA, Suelly Maria Mendes RIBEIRO, Samuel de Carvalho CHAVES, Flavia ARTESE, David NORMANDO

**Affiliations:** 1 Department of Orthodontics, Dental School, Federal University of Pará (UFPA, Belém, Pará, Brazil).; 2 Department of Pediatric Dentistry, Dental School, Federal University of Pará (UFPA, Belém, Pará, Brazil).; 3 Department of Orthodontics, Dental School, Rio de Janeiro State University (UERJ, Rio de Janeiro, Brazil).

**Keywords:** Open bite, Interceptive orthodontics, Quality of life

## Abstract

**Introduction:**

The use of lingual spurs has been described as one efficient option, with great stability of results, but with scarce information of toleration for use in the mixed and permanent dentition phases.

**Objective:**

The purpose of this study was to assess the impact of lingual spurs on the oral health-related quality of life of children and/or adolescents during anterior open bite treatment.

**Methods:**

The review was recorded in the PROSPERO database. Eight electronic databases and partial gray literature were searched, without restrictions until march 2022. A manual search was also performed in the references of the included articles. Studies assessing the impact of lingual spurs on the oral health-related quality of life were included. Risk of bias was assessed using JBI or ROBINS-I tool, according to the study design. The level of evidence was assessed through GRADE.

**Results:**

Five studies met the eligibility criteria. Two non-randomized clinical trials had a serious risk of bias. Of the case-series studies, two had a low risk of bias and the other, a moderate risk of bias. The certainty of the evidence was classified as very low for all the evaluated results. In general, the studies reported an initial negative impact with the use of lingual spurs, however this was transitory in nature. A quantitative analysis was not performed due to the great heterogeneity between the studies.

**Conclusion:**

Current evidence, although limited, suggests that lingual spurs have an initial transient negative impact during interceptive treatment. Additional well-conducted randomized clinical trials are needed.

## RESUMO

**Introdução:**

O uso de esporões linguais tem sido descrito como uma opção eficiente, com grande estabilidade de resultados, mas com poucas informações quanto à tolerância de seu uso nas fases de dentição mista e permanente.

**Objetivo:**

O objetivo deste estudo foi avaliar o impacto dos esporões linguais na qualidade de vida relacionada à saúde bucal de crianças e/ou adolescentes durante o tratamento da mordida aberta anterior.

**Métodos:**

Essa revisão foi registrada no banco de dados PROSPERO. Oito bases de dados eletrônicas e parte da literatura cinzenta foram pesquisadas, sem restrições, até março de 2022. Uma busca manual também foi realizada nas referências dos artigos incluídos. Estudos avaliando o impacto dos esporões linguais na qualidade de vida relacionada à saúde bucal foram incluídos. O risco de viés foi avaliado usando a ferramenta JBI ou ROBINS-I, de acordo com o desenho do estudo. O nível de evidência foi avaliado por meio do GRADE.

**Resultados:**

Cinco estudos preencheram os critérios de elegibilidade. Dois ensaios clínicos não randomizados apresentaram sério risco de viés. Dos estudos de série de casos, dois tiveram baixo risco de viés e o outro, risco moderado de viés. A certeza da evidência foi classificada como muito baixa para todos os resultados avaliados. Em geral, os estudos relataram um impacto negativo inicial com o uso dos esporões linguais; porém, de caráter transitório. Não foi realizada análise quantitativa, devido à grande heterogeneidade entre os estudos.

**Conclusão:**

As evidências atuais, embora limitadas, sugerem que os esporões linguais têm um impacto negativo transitório inicial durante o tratamento interceptativo. São necessários mais ensaios clínicos randomizados bem conduzidos.

## INTRODUCTION

Anterior open bite can have a significant impact on the quality of life in children and adolescents, due to the severe aesthetic-functional impairment,^1^ ^,^ ^2^ and the orthodontic treatment is able to improve quality of life in such patientes.^2^ However, long-term treatment stability can be a challenge.^1^ This is probably due to the difficulty in recognizing the multifactorial aspect of the etiology of anterior open bites, which may include deleterious habits and oral breathing, vertical growth pattern, abnormal size and incorrect tongue function^3^. Previous studies have correlated incorrect tongue posture as the main risk factor for relapse.^4^ ^,^ ^5^

Although several approaches regarding anterior open bite treatment are available, there is still no consensus on which therapy would be able to control in the long term the oral dysfunctions and myoskeletal problems present in this malocclusion.^6^ Among the options, lingual spurs is one approach that uses intraoral devices.^6^ They serve as a reminder for the patient to interrupt tongue posture habits, promoting postural training of the tongue due to the triggering of nociceptive or proprioceptive reflexes, generating a positive effect in anterior open bite treatment and providing good clinical results.^6^ ^,^ ^7^ However, some orthodontists are cautious with the indication of spurs, due to possible physical and psychological negative reactions of the child. They pierces the tongue, providing painful feedback, and can be seen as punitive structures, inflicting pain and suffering disproportionate to the needs of the patient.^8^

Some systematic reviews have evaluated the efficiency of different early treatment protocols used to correct anterior open bite.^9^ ^,^ ^10^ However, there is no systematic analysis of the evidence on the impact of lingual spurs on quality of life. Accordingly, the aim of this review was to investigate the impact on the quality of life, in children and adolescents, of using spurs for anterior open bite treatment.

## MATERIAL AND METHODS

### PROTOCOL AND REGISTRATION

This systematic review was registered at the PROSPERO database (CRD42020203780) and performed according to PRISMA (Preferred Reporting Items for Systematic Review and Meta-Analysis) guidelines.^11^

### ELIGIBILITY CRITERIA

The following selection criteria were adopted:

Study design: prospective or retrospective studies.
Population: children and/or adolescents (4 to 18 years).
Intervention: lingual or palatal spurs.
Comparison: untreated population or other interceptive appliances as control group, or cases series.
Outcome: impact of lingual spurs on the oral health-related quality of life (functional and psychosocial outcomes of oral disorders).
Exclusion criteria: Animal or laboratory studies, technical articles, case reports and literature reviews.

### INFORMATION SOURCES

The following databases were searched: PubMed, Scopus, Web of Science, Cochrane Library, LILACS and ClinicalTrials. Grey literature was consulted through OpenGrey and Google Scholar. A hand search was conducted by reading the references of the included articles, for eventual additional relevant studies. No restriction on language or date of publication was applied. The search was continued until March 15th, 2022.

### SEARCH STRATEGY AND STUDY SELECTION

The databases were independently searched by two reviews (LBM and SMMR). Disagreements were settled by discussion and consensus and, when necessary, a third author’s opinion (SCCJ) was consulted. The search strategy was developed through a combination of Mesh, entry terms and keywords related to the PICO strategy using Boolean operators (Appendix 1).

After the searches, the results were imported to a reference manager software (EndNote, x9 version; Clarivate Analytics, Philadelphia, PA). Duplicate studies were excluded by automatic and manual assessment. The selection process was performed in two phases. In the first phase, the title and abstract that did not follow the established eligibility criteria were excluded. In the second phase, the articles remaining from phase I were assessed by reading the full-text. In addition, the reference list of the selected studies were also evaluated to retrieve new articles that followed the eligibility criteria.

### DATA ITEMS

Data collected from each article included: authors, year of publication, country, study design, participants, age, follow-up, statistical analysis, methods of evaluation and results (Table 1). 

**Table 1:** Data summary of the studies included in this review.

Author, year (study design)	Participants (n)	Age (years)	Follow-up	Statistics Analysis	Methods of Evaluation	Results
Canuto et al.^15^ 2016 (NRCT* - Prospective)	Bonded lingual spurs: 20 Conventional spurs: 21 Untreated subjects: 27	7.6 - 10.8	12 months	x^2^ test	Questionnaire, similar to that proposed by McRae^17^, 2010	Comparative acceptance evaluation showed both appliances were well tolerated: p=0.30 Discomfort time was at most 7 days with both appliances: p=0.37 Bonded lingual spurs had better acceptance than conventional spurs during chewing and eating: p=0.015
Haryett et al.^16^ 1970 (NRCT* - Retrospective)	Untreated subjects: 8 Palatal crib with spurs: 11 Palatal cribs with spurs and psychological treatment:10 Crib without spurs: 27 Crib without spurs and psychological treatment: 10	≥ 4	20 months	x^2^ test	Interviews with parents and rating scales	Irritability with spurs: 25% Irritation of the palate with spurs: 31%; Some speech difficulty with spurs: 66% Some difficulty in eating with spurs: 50% Sleep disturbance with spurs: 50%; Restlessness: 81%
McRae^17^ 2010 (Case-series)	Bonded lingual spurs n=12 9 females and 3 males	7.1 - 17.2	6 months	paired-sample Sign Test	Questionnaire developed by authors	Some minimal initial discomfort and this observation did not change substantially over time (p=0.969) After only one month of therapy, the spurs were rated as either easy or neutral to tolerate in all categories except eating and tongue pain (p<0.05) Two droup-out in follow up
Araújo et al.^18^ 2011 (Case-series)	Conventional spurs ≤ 14 years: 33 Conventional spurs ≥ 15 years: 39	10.4 - 16.8	8.1 months	x^2^ test and Fisher’s exact test	Questionnaire developed by orthodontists, physiologists and psychologists	Accepted the treatment: 98.6% Aggressive: 58.2% Felt some degree of pain: 86.1% Discomfort and pain may continue up as 10 days: 92.0% Female group demonstrated a higher tolerance (p<0.05) Speech and chewing impairments were the most frequent functional problem: 79.2% Sleep disturbances: 8.3%
Moda^19^ 2020 (Case-series)	Bonded lingual spurs (8-10 years): 9 Bonded lingual spurs (11-14 years): 7	8.5 - 12.8	3 months	Friedman’s test and Wilcoxon Signed Rank test	**CPQ_8-10_, CPQ_11-14_ and Pain rating Scale Wong-Baker Faces	Greatest impact on oral symptoms before the placement of spurs: p=0.01 The scores had a decreasing trend for oral symptoms and functional limitations over time: p=0.04 No pain perception on the Wong-Baker faces pain scale: p>0.05 One droup-out in follow-up

*NRCT = Nonrandomized clinical trials; **CPQ - Child Perception Questionnaire.

### RISK OF BIAS ASSESSMENT

For the case-series, the risk of bias was performed following the Joanna Briggs Institute (JBI) Critical Appraisal Checklist tool.^12^ The checklist for case-series studies uses ten criteria. Each component was rated “yes”, “no”, “unclear”, or “not applicable”. With 1-3 “yes” scores, the risk of bias classification is high; 4-6 “yes” scores, the risk is moderate and 7-10 scores, there is low risk of bias (Table 2).

**Table 2:** Risk of bias in selected case-series.

Questions/Author	MacRae^17^, 2010	Araújo et al.^18^, 2011	Moda^19^, 2020
Were there clear criteria for inclusion in the case series?	Yes	Unclear	Yes
Was the condition measured in a standard and reliable way for all participants included in the case series?	Yes	Yes	Yes
Were valid methods used for identification of the condition for all participants included in the case series?	Unclear	Yes	Yes
Did the case series have consecutive inclusion of participants?	Yes	Unclear	Yes
Did the case series have complete inclusion of participants?	Yes	Unclear	Yes
Was there clear reporting of the demographics of the participants in the study?	Yes	Yes	Yes
Was there clear reporting of clinical information of the participants?	Yes	Yes	Yes
Were the outcomes or follow-up results of cases clearly reported?	Unclear	Yes	Unclear
Was there clear reporting of the presenting site(s)/clinic(s) demographic information?	Yes	Unclear	Yes
Was statistical analysis appropriate?	No	No	Yes
Risk of bias	Low	Moderate	Low

The ROBINS-I tool Risk of Bias in Non-Randomized Studies of Interventions)^13^ was used in nonrandomized studies. This checklist presents three main evaluation domains. The risk of bias was assessed for each domain and classified as “low”, “moderate”, “serious”, “critical” or “no information” (Table 3). Each analysis was made by two authors (LBM and SMMR), and disagreements were solved by a third reviewer (SCCJ).

**Table 3:** Risk of bias in nonrandomized selected studies.

Domains/ROBINS-I Tool								
	Pre-intervention		Intervention		Post-intervention			
Author	Bias due to confounding	Bias in selecting participants for study	Bias in classifying Interventions	Bias due to deviations from intended intervention	Bias due to missing data	Bias in measuring outcomes	Bias in selecting reported result	Overall risk of bias
Canuto^15^, 2016	Moderate	Serious	Low	Low	Low	Low	Moderate	Serious risk of bias
Harryet, 1970^16^	Moderate	Moderate	Low	Moderate	Low	Low	Serious	Serious risk of bias

### LEVEL OF EVIDENCE

The included articles were given a narrative score related to the outcome assessed in this review (i.e. the impact of lingual spurs on the oral health-related quality of life of children and/or adolescents during anterior open bite treatment) according to the GRADE tool (Grading of Recommendations, Assessment, Development and Evaluation).^14^ This tool considered five aspects for rating the quality of evidence as high, moderate, low or very low.

### SYNTHESIS OF METHODS

The results are provided in a narrative synthesis of the included studies that comprised study type, sample size, age of population, intervention group, comparison group and outcome.

## RESULTS

### STUDY SELECTION

**Figure 1:**
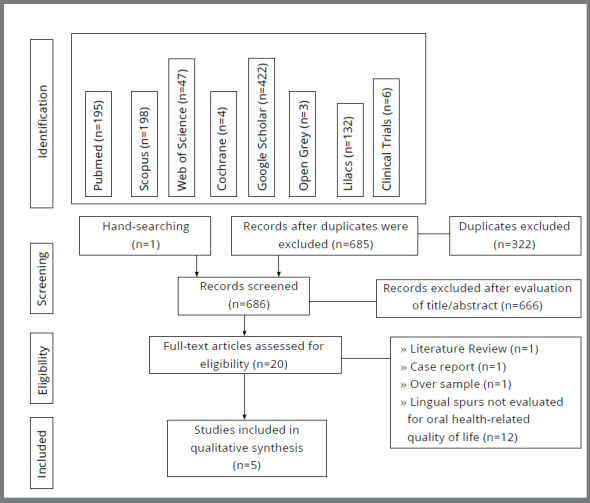
Study identification flow diagram.

The electronic search revealed a total of 1,007 citations: 195 from PubMed, 198 from SCOPUS, 47 from Web of Science, 4 from Cochrane, 132 from LILACS, 422 from Google Scholar, 6 from Clinical Trials, and 3 from OpenGrey. After removing duplicates, 685 studies remained. One study was added for screening after a hand search, resulting in 686 articles for review. After reading the titles and abstracts, 20 articles were evaluated in full, and 15 were excluded. The reasons for exclusion are show in Table 4. As a result, 5 articles were included^15^ ^-^ ^19^ (Fig 1).

**Table 4:** List and reasons for excluded studies.

Reference	Reasons for exclusion
Harryet (1967)	Over sample
Justus (2001)	Literature review
Meyer-Marcotty et al. (2007)	Lingual spurs not evaluated for oral health-related quality of life
Cassis et al. (2010)	Lingual spurs not evaluated for oral health-related quality of life
Cassis et al. (2012)	Lingual spurs not evaluated for oral health-related quality of life
Benjamin (2013)	Lingual spurs not evaluated for oral health-related quality of life
Meyer-Marcotty et al. (2013)	Lingual spurs not evaluated for oral health-related quality of life
Urnau (2014)	Lingual spurs not evaluated for oral health-related quality of life
Insabralde et al. (2016)	Lingual spurs not evaluated for oral health-related quality of life
Leite et al. (2016)	Lingual spurs not evaluated for oral health-related quality of life
Dias (2017)	Lingual spurs not evaluated for oral health-related quality of life
Rossato et al. (2018)	Lingual spurs not evaluated for oral health-related quality of life
Cassis et al. (2018)	Lingual spurs not evaluated for oral health-related quality of life
Dias et al. (2019)	Case report
Rossato (2019)	Lingual spurs not evaluated for oral health-related quality of life

### STUDY CHARACTERISTICS

The characteristics of the included studies are described in Table 1. Selected studies were published between 1970 and 2020.^15^ ^-^ ^19^ Two studies^15^ ^,^ ^16^ were nonrandomized trials (one was prospective^15^ and the other was retrospective^16^), and three studies were case-series.^17^ ^-^ ^19^ The follow-up period ranged from 3 months^19^ to 20 months.^16^ The sample size ranged from 12^17^ to 72 individuals^18^ The average patient age was from 4 to 17 years among the studies.^15^ ^-^ ^19^ Only one study^16^ did not describe the mean age of patients. Both sexes were included.^15^ ^-^ ^19^

The methods used to evaluate the impact of lingual spurs on the oral health-related quality of life of children and/or adolescents during anterior open bite treatment were questionnaires^15^ ^-^ ^19^ and pain scales.^16^ ^,^ ^19^ Three studies used their own questionnaires developed for their research.^15^ ^,^ ^17^ ^,^ ^18^ One study conducted interviews with parents or guardians with a rating scale.^16^ It is important to highlight that only one study^19^ used a pain scale and validated questionnaires to assess the repercussions of oral health problems on the quality of life of children.

In relation to lingual spur types, two studies bonded lingual spurs to the palatal and lingual surfaces of the maxillary and mandibular incisors^17^ ^,^ ^19^. In one study, treatment consisted of a mandibular lingual arch and spurs^18^. In another, two different types of spurs were used: bonded lingual spurs, compared with conventional spurs^15^. In a fifth study, a palatal crib with spurs was used.^16^

### RESULTS OF INDIVIDUAL STUDIES

The use of lingual spurs in early treatment for anterior open bite has some initial negative impacts on the oral health-related quality of life, the average open bite treatment duration was between 3 to 12 months. In the studies, there was the application of the questionnaire during^15^ and before and during treatment.^17^ ^,^ ^18^ ^,^ ^19^

There was a questionnaire application during treatment with 4 objective questions for speech, feeding, tongue pain and discomfort, and use of spurs. The spurs were well-tolerated after 7 days during the functions of chewing and feeding.^15^ After a psychological evaluation, the results include a temporary period of disturbance, difficulty in speech, and some difficulty in eating, ranging from 1 day to 3 months.^16^ In this study, the difficulties were in the categories of speech, feeding, aesthetics, and tongue pain at the beginning and end of treatment. The spurs were well-tolerated by all individuals, classified as ‘easy’ and ‘neutral’ in all categories, except for feeding and tongue pain. In the ‘aesthetics’ category, they were all scored as ‘easy’.^17^ Among the categories researched, the responses differed between the groups studied, for behavior change, acceptance of treatment, duration of pain during treatment, and change in the function of chewing.^18^ In this study there was a decreasing trend of oral symptoms and functional limitations over time, being the greatest impact on the domains evaluated before lingual spurs bonding.^19^

Oral symptoms reported were pain in teeth, bad breath, mouth sores and food caught between teeth,^19^ palate irritation^16^ and tongue pain.^17^ ^,^ ^18^ However, this discomfort, when assessed through questionnaires and pain scales, seems tolerable and temporary, tending to decrease over time. Yet, regarding the recorded discomfort, the findings were present for a maximum of 7^15^ ^,^ ^19^ to 10 days^18^ in most patients. Throughout treatment only two studies reported losses of participants: two losses^17^ and one loss.^19^

Speech and chewing problems were the most common functional complications developed during lingual spur therapy;^15^ ^-^ ^19^ and these were also reported as decreasing over time. Sleep disorders such as restlessness and nocturnal enuresis were also reported in a transient manner.^16^ One study reported greater acceptance of bonded lingual spurs, compared to conventional spurs.^15^

One study concluded that treatment with spurs does not seem to be related to the development of other parafunctional habits, such as nail biting, body scratching, nibbling hair or clothes and snapping fingers. Nevertheless, patients became more restless, bored and they cried more easily.^16^

### SYNTHESIS OF RESULTS

A meta-analysis was not considered in this systematic review due to the methodological heterogeneity. The included studies used different design of appliances and methods to evaluate oral health-related quality of life.

### BIAS RISK ASSESSMENT

Regarding the case-series studies, two resulted in a low risk of bias^17^ ^,^ ^19^ and one in a moderate risk.^18^ In one of the studies, the instrument used to measure quality of life was not validated. In addition, there was a large difference in proportion between genders, the authors used inadequate statistical tests, which may induce to some bias, and the follow-up results were not clear.^17^ Another study^18^ used a convenience sample, determining its allocation through the participants’ date of birth. The instrument used for assessing the impact on quality of life was developed by orthodontists, physiologists and psychologists, and the authors did not clearly report the inclusion criteria. After email contact, the authors clarified that they established a division by age and psychological criteria.

 In addition, there is heterogeneity between the studied groups, which can generate greater variability.^18^ In the third study, the authors did not clearly report the outcomes or results of the follow-up period.^19^

Regarding the non-randomized clinical studies,^15^ ^,^ ^16^ both presented serious risks of bias. In one study, the authors used a non-validated instrument to measure oral health-related quality of life, with questions created by the authors themselves. They did not perform a sample size calculation, and used inadequate statistical tests, which may induce an important measurement bias. In addition, they presented retrospective definitions of some assigned aspects of interventions.^16^ The other study also used a non-validated instrument to measure quality of life^15^ adapted from a previous study.^17^ The researchers determined a rule of deterministic attribution as a way of trying to guarantee an exact proportion between the groups, alternating the records received from each patient, which can generate important selection bias. In addition, the control group was compared with different subjects from the experimental groups.^15^

Blinding was not considered a determining factor for the analysis of risk of bias in relation to the research topic. The adaptation and assessment of lingual spurs requires visual clinical monitoring, which does not allow the blinding of participants and operators. The risk of bias assessments for all included studies are shown in Tables 2 and 3.

### LEVEL OF EVIDENCE

The level of certainty of outcomes evaluated in this systematic review were classified as “very low”^15^ ^-^ ^19^ due to limitations in the study design,^15^ ^-^ ^19^ great heterogeneity in the samples^17^ ^,^ ^18^ and lack of clarity as to the outcomes or results of follow up.^17^ ^,^ ^19^ Therefore, confidence in the estimate of the effect is limited. In this way, there is a possibility that the real effect is substantially different (Table 5).

**Table 5:** Grading of Recommendation, Assessment, Development, and Evaluation (GRADE) instrument.^14^

Certainty assessment							Impact	Certainty	Importance
№ of studies	Study design	Risk of bias	Inconsistency	Indirectness	Imprecision	Other considerations			
Oral health-related quality of life (rated with: questionnaires )									
2	NRCT (1 prospective and 1 retrospective)	Serious^a^	Serious^b^	Not serious	Not serious	Highly suspicious publication bias^c^	Both evaluated articles showed some negative impact on the quality of life related to the oral health of children and/or adolescents. Discomfort, speech and chewing problems were the most common changes, with transitory nature.	⨁◯◯◯ VERY LOW	CRITICAL
3	Case-series	Serious^d^	Serious^b^	Not serious	Not serious	Highly suspicious publication bias^e^	Of the three studies, two showed an initial negative impact of a transitory nature. Except for one, who completed minimal initial discomfort with no changes over time. Discomfort, speech problems and chewing were the most reported oral symptoms.	⨁◯◯◯ VERY LOW	IMPORTANT

CI = Confidence interval.

NRCT = Nonrandomized clinical trials.

a Two studies showed serious ROBINS. ^b^ There is some heterogeneity in the study sample. ^c^ Not all reported results corresponded to all intended. ^d^ One study had no clear inclusion criteria. ^e^ The outcomes or follow up results were not clearly reported.

## DISCUSSION

### SUMMARY OF EVIDENCE

Among the five studies included in this review, all described that the physical and psychological negative reactions found during the lingual spur treatment were of a transitory nature. There was a tendency for these reactions to decrease throughout treatment and were tolerated by patients, with a range of 7,^15^ ^-^ ^19^ 15 to 20 days of adaptability.^17^ ^,^ ^18^ The evaluated studies were characterized as two prospective^15^ and retrospective^16^ non-randomized clinical studies, and three case-series.^17^ ^-^ ^19^ Two studies had a serious risk of bias^15^ ^,^ ^16^, two others had a low risk^17^ ^,^ ^19^ and one, a moderate risk.^18^

The impact of lingual spurs on children’s oral health-related quality of life may have been influenced by some factors, such as different perceptions between genders. One study observed that this sensation was more tolerated by girls.^18^ However, the painful perception can have a biological influence among children eight years or older, as boys tend to be reluctant to express emotions related to pain,^20^ so these influences must be considered.

Oral speech and chewing functions, previously impaired by the presence of an open bite, were evaluated in the five studies.^15^ ^-^ ^19^ The findings showed that the presence of spurs altered speech at the beginning of treatment, but it was readjusted within a maximum of 3 weeks,^16^ with greater perception in older children^18^. What the authors seem to agree on is that speech was substantially improved after treatment with spurs and, consequently, open bite closure, corroborating the findings in the literature.^21^ ^,^ ^22^

The effects on chewing due to the use of spurs were also transitory, according to the authors. However, the adaptation period was slightly longer, about 30 days,^17^ and younger children had greater perception.^18^ Numerous physiological factors can influence chewing, such as the number and type of teeth, and these can change with children’s age. These changes can influence the stabilization and occlusion of the jaw and, thus, the chewing function of younger children.^22^

Other negative impacts that were reported were that children became more upset, irritated and cried more easily, ranging from 1 to 30 days, ceasing in 1 to 3 weeks.^16^ In addition, they had temporary sleep disturbances and became more restless.^16^ This observation of emotional disorders may be associated with fear and anxiety of dental treatment. There is evidence that psychological aspects influence the patient’s perception of dental care, so that the patient’s level of anxiety, state of attention and emotions can make them overestimate the pain they will feel.^23^

About the impact of patient losses during follow-up on the result, it is known that it is important to consider all individuals included in the sample and not just those who completed the entire follow-up period. However, studies suggest that the impact of the loss depends on the number of individuals who abandon or are excluded.^24^ Although there is no established limit from which there would be a significant compromise in the results, it is suggested that studies with loss of patients above 20% should not be accepted.^24^ In this review, only two studies reported losses,^17^ ^,^ ^19^ one loss in one study^19^ and two in another.^17^

Regarding the positive impacts, studies suggest the advantage of spurs, as it is a fixed device, which does not depend on the patient’s collaboration, it is quick to install, can be used in both the upper and lower arches at a low cost.^25^ In addition, they were considered good aesthetic options.^15^ It is recognized that facial appearance plays an important role in the judgment of personal attractiveness and also in the development of self-esteem.^26^

Understanding the importance of this subject for further clinical clarification, a randomized clinical trial was found in progress, while searching the databases of clinical trial records. However, to date, the study has not been published or any results have been reported.^27^

### LIMITATIONS/RECOMMENDATIONS

The case-series^17^ ^-^ ^19^ and the non-randomized clinical studies^15^ ^,^ ^16^ included in this review had some limitations in their methods and study design, which impacted their risk of bias assessment.

The variation in the methods of assessing oral health-related quality of life may have been a confusing factor for the results found in the studies included in this review, given that there was no homogeneity in the choice of the questionnaires used.^15^ ^-^ ^19^ Biases related to questionnaire-based studies are common, since the results depend of the honesty of the patient and the accuracy of their responses. In addition, it should be taken into account that children can adapt or get used to their health conditions over time and can respond with lower impact scores when a questionnaire is reapplied later.^28^

Still, the lack of data on dropouts could have some influence on the result of the impact of the perception of spurs related to quality of life^17^ ^,^ ^19^. Losses of patient during the study can affect the conclusions, since the unknown response of these patients to treatment may change the results of the comparison.^24^

Of the five studies, only one^19^ used a validated questionnaire for this purpose. The importance of investigating this issue more precisely is known, which is using valid and reliable tools to obtain consistent information to provide additional data for making clinical decisions or assessing treatment success.^29^ ^,^ ^30^

The lack of standardization and other important methodological limitations of the studies included in this review show the need for future standardized clinical studies regarding methodology and error analysis. In addition, further studies with longer follow-up periods are needed. Therefore, a RCT evaluating the impact of lingual spurs on oral health-related quality of life is mandatory.

## CONCLUSION

Current evidence points out that the anterior open bite treatment with lingual spurs causes negative impacts on the oral health-related quality of life, more specifically discomfort in speech and chewing, but these impacts seem to be transitory by nature. These results should be evaluated with caution, based on the low level of certainty, suggesting the need for new well-designed studies.

## Appendix 1: Database and Search strategies.

[Table t1app]

**Table t1app:**

Database	Keywords	Results
Pubmed	((((((((((((((((((((((((((((((((((((((((((((((((((((((((Infant[MeSH Terms]) OR (Infant[Title/Abstract])) OR (Toddler[Title/Abstract])) OR (Pediatrics[MeSH Terms])) OR (Pediatrics[Title/Abstract])) OR (Pediatric[Title/Abstract])) OR (Paediatric[Title/Abstract])) OR (Child[MeSH Terms])) OR (Child[Title/Abstract])) OR (Children[Title/Abstract])) OR (Minors[MeSH Terms])) OR (Minors[Title/Abstract])) OR (Minor[Title/Abstract])) OR (Adolescent[MeSH Terms])) OR (Adolescent[Title/Abstract])) OR (Adolescents[Title/Abstract])) OR (Adolescence[Title/Abstract])) OR (Teens[Title/Abstract])) OR (Teen[Title/Abstract])) OR (Teenagers[Title/Abstract])) OR (Teenager[Title/Abstract])) OR (Youth[Title/Abstract])) OR (Youths[Title/Abstract])) OR (Adolescents, Female[Title/Abstract])) OR (Adolescent, Female[Title/Abstract])) OR (Female Adolescent[Title/Abstract])) OR (Female Adolescents[Title/Abstract])) OR (Adolescents, Male[Title/Abstract])) OR (Adolescent, Male[Title/Abstract])) OR (Male Adolescent[Title/Abstract])) OR (Male Adolescents[Title/Abstract])) OR (Boy[Title/Abstract])) OR (Boys[Title/Abstract])) OR (Girl[Title/Abstract])) OR (Girls[Title/Abstract])) OR (Schools, Nursery[MeSH Terms])) OR (Schools, Nursery[Title/Abstract])) OR (Nursery schools[Title/Abstract])) OR (Nursery school[Title/Abstract])) OR (School, Nursery[Title/Abstract])) OR (Child, Preschool[MeSH Terms])) OR (Child, Preschool[Title/Abstract])) OR (Preschool Child[Title/Abstract])) OR (Children, Preschool[Title/Abstract])) OR (Preschool Children[Title/Abstract])) OR (Day care[Title/Abstract])) OR (Kindergarten[Title/Abstract])) OR (Kindergarden[Title/Abstract])) OR (Elementary school[Title/Abstract])) OR (Schoolchild[Title/Abstract])) OR (Middle school[Title/Abstract])) OR (High school[Title/Abstract]))) AND ((((((((((Open bite[MeSH Terms]) OR (Open bite[Title/Abstract])) OR (Bite, Open[Title/Abstract])) OR (Nonoclusion[Title/Abstract])) OR (Openbite[Title/Abstract])) OR (Apertognathia[Title/Abstract])) OR (Anterior open bite[Title/Abstract])) OR (Anterior open-bite[Title/Abstract])) OR (Open-bite[Title/Abstract]))))) AND (((((((((((((((((((((((((((((((((((((((((((((((((((((Orthodontics, Interceptive[MeSH Terms]) OR (Orthodontics, Interceptive[Title/Abstract])) OR (Interceptive orthodontics[Title/Abstract])) OR (Orthodontic Appliances, Functional[MeSH Terms])) OR (Orthodontic Appliances, Functional[Title/Abstract])) OR (Appliance, Functional Orthodontic[Title/Abstract])) OR (Appliances, Functional Orthodontic[Title/Abstract])) OR (Functional Orthodontic Appliance[Title/Abstract])) OR (Functional Orthodontic Appliance[Title/Abstract])) OR (Orthodontic Appliance, Functional[Title/Abstract])) OR (Early orthodontic treatment[Title/Abstract])) OR (Early open bite treatment[Title/Abstract])) OR (Anterior open bite treatment[Title/Abstract])) OR (Orthopaedic treatment[Title/Abstract])) OR (Habit appliances[Title/Abstract])) OR (Fixed intraoral habit appliance[Title/Abstract])) OR (Spur[Title/Abstract])) OR (Spurs[Title/Abstract])) OR (Spur therapy[Title/Abstract])) OR (Spur appliance[Title/Abstract])) OR (Sharp spur[Title/Abstract])) OR (Sharp spurs[Title/Abstract])) OR (Tongue spur[Title/Abstract])) OR (Tongue spurs[Title/Abstract])) OR (Lingual spurs[Title/Abstract])) OR (Lingual spur[Title/Abstract])) OR (Palatal spur[Title/Abstract])) OR (Palatal spurs[Title/Abstract])) OR (Spike[Title/Abstract])) OR (Spikes[Title/Abstract])) OR (Lingual spike[Title/Abstract])) OR (Lingual spikes[Title/Abstract])) OR (Bonded spur[Title/Abstract])) OR (Bonded spurs[Title/Abstract])) OR (Spur bonded[Title/Abstract])) OR (Spurs bonded[Title/Abstract])) OR (Bonded lingual spur[Title/Abstract])) OR (Bonded lingual spurs[Title/Abstract])) OR (Bonded palatal spur[Title/Abstract])) OR (Bonded palatal spurs[Title/Abstract])) OR (Bondable lingual spur[Title/Abstract])) OR (Bondable lingual spurs[Title/Abstract])) OR (Bondable lingual tongue spur[Title/Abstract])) OR (Bondable lingual tongue spurs[Title/Abstract])) OR (Conventional spur[Title/Abstract])) OR (Conventional spurs[Title/Abstract])) OR (Conventional orthodontic spur[Title/Abstract])) OR (Conventional orthodontic spurs[Title/Abstract])) OR (Conventional lingual spur[Title/Abstract])) OR (Conventional lingual spurs[Title/Abstract])) OR (Banded spur[Title/Abstract])) OR (Banded spurs[Title/Abstract]))))	195
Scopus	(((TITLE-ABS-KEY(Infant*) OR TITLE-ABS-KEY(“Toddler”) OR TITLE-ABS-KEY(Pediatrics*) OR TITLE-ABS-KEY(“Pediatric”) OR TITLE-ABS-KEY(“Paediatric”) OR TITLE-ABS-KEY(Child*) OR TITLE-ABS-KEY(“Children”) OR TITLE-ABS-KEY(Minors*) OR TITLE-ABS-KEY(“Minor”) OR TITLE-ABS-KEY(Adolescent*) OR TITLE-ABS-KEY(“Adolescents”) OR TITLE-ABS-KEY(“Adolescence”) OR TITLE-ABS-KEY(“Teens”) OR TITLE-ABS-KEY(“Teen”) OR TITLE-ABS-KEY(“Teenagers”) OR TITLE-ABS-KEY(“Teenager”) OR TITLE-ABS-KEY(“Youth”) OR TITLE-ABS-KEY(“Youths”) OR TITLE-ABS-KEY(“Female Adolescent”) OR TITLE-ABS-KEY(“Female Adolescents”) OR TITLE-ABS-KEY(“Male Adolescent”) OR TITLE-ABS-KEY(“Male Adolescents”) OR TITLE-ABS-KEY(“Boy”) OR TITLE-ABS-KEY(“Boys”) OR TITLE-ABS-KEY(“Girl”) OR TITLE-ABS-KEY(“Girls”) OR TITLE-ABS-KEY(Schools Nursery*) OR TITLE-ABS-KEY(“Nursery schools”) OR TITLE-ABS-KEY(“Nursery school”) OR TITLE-ABS-KEY(Child Preschool*) OR TITLE-ABS-KEY(“Preschool Child”) OR TITLE-ABS-KEY(“Preschool Children”) OR TITLE-ABS-KEY(“Day care”) OR TITLE-ABS-KEY(“Kindergarten”) OR TITLE-ABS-KEY(“Kindergarden”) OR TITLE-ABS-KEY(“Elementary school”) OR TITLE-ABS-KEY(“Schoolchild”) OR TITLE-ABS-KEY(“Middle school”) OR TITLE-ABS-KEY(“High school”))) AND ((TITLE-ABS-KEY(Open bite*) OR TITLE-ABS-KEY(“Nonoclusion”) OR TITLE-ABS-KEY(“Openbite”) OR TITLE-ABS-KEY(“Apertognathia”) OR TITLE-ABS-KEY(“Anterior open bite”) OR TITLE-ABS-KEY(“Anterior open-bite”) OR TITLE-ABS-KEY(“Open-bite”)))) AND ((TITLE-ABS-KEY(Orthodontics Interceptive*) OR TITLE-ABS-KEY(“Interceptive orthodontics”) OR TITLE-ABS-KEY(Orthodontic Appliances Functional*) OR TITLE-ABS-KEY(“Functional Orthodontic Appliance”) OR TITLE-ABS-KEY(“Functional Orthodontic Appliance”) OR TITLE-ABS-KEY(“Early orthodontic treatment”) OR TITLE-ABS-KEY(“Early open bite treatment”) OR TITLE-ABS-KEY(“Anterior open bite treatment”) OR TITLE-ABS-KEY(“Orthopaedic treatment”) OR TITLE-ABS-KEY(“Habit appliances”) OR TITLE-ABS-KEY(“Fixed intraoral habit appliance”) OR TITLE-ABS-KEY(“Spur”) OR TITLE-ABS-KEY(“Spurs”) OR TITLE-ABS-KEY(“Spur therapy”) OR TITLE-ABS-KEY(“Spur appliance”) OR TITLE-ABS-KEY(“Sharp spur”) OR TITLE-ABS-KEY(“Sharp spurs”) OR TITLE-ABS-KEY(“Tongue spur”) OR TITLE-ABS-KEY(“Tongue spurs”) OR TITLE-ABS-KEY(“Tongue spur”) OR TITLE-ABS-KEY(“Lingual spurs”) OR TITLE-ABS-KEY(“Lingual spur”) OR TITLE-ABS-KEY(“Palatal spur”) OR TITLE-ABS-KEY(“Palatal spurs”) OR TITLE-ABS-KEY(“Spike”) OR TITLE-ABS-KEY(“Spikes”) OR TITLE-ABS-KEY(“Lingual spike”) OR TITLE-ABS-KEY(“Lingual spikes”) OR TITLE-ABS-KEY(“Bonded spur”) OR TITLE-ABS-KEY(“Bonded spurs”) OR TITLE-ABS-KEY(“Spur bonded”) OR TITLE-ABS-KEY(“Spurs bonded”) OR TITLE-ABS-KEY(“Bonded lingual spur”) OR TITLE-ABS-KEY(“Bonded lingual spurs”) OR TITLE-ABS-KEY(“Bonded palatal spur”) OR TITLE-ABS-KEY(“Bonded palatal spurs”) OR TITLE-ABS-KEY(“Bondable lingual spur”) OR TITLE-ABS-KEY(“Bondable lingual spurs”) OR TITLE-ABS-KEY(“Bondable lingual tongue spur”) OR TITLE-ABS-KEY(“Bondable lingual tongue spurs”) OR TITLE-ABS-KEY(“Conventional spur”) OR TITLE-ABS-KEY(“Conventional spurs”) OR TITLE-ABS-KEY(“Conventional orthodontic spur”) OR TITLE-ABS-KEY(“Conventional orthodontic spurs”) OR TITLE-ABS-KEY(“Conventional lingual spur”) OR TITLE-ABS-KEY(“Conventional lingual spurs”) OR TITLE-ABS-KEY(“Banded spur”) OR TITLE-ABS-KEY(“Banded spurs”)))	198
Web of Science	TÓPICO: (Infant*) OR TÓPICO: (“Toddler”) OR TÓPICO: (Pediatrics*) OR TÓPICO: (“Pediatric”) OR TÓPICO: (“Paediatric”) OR TÓPICO: (Child*) OR TÓPICO: (“Children”) OR TÓPICO: (Minors*) OR TÓPICO: (“Minor”) OR TÓPICO: (Adolescent*) OR TÓPICO: (“Adolescents”) OR TÓPICO: (“Adolescence”) OR TÓPICO: (“Teens”) OR TÓPICO: (“Teen”) OR TÓPICO: (“Teenagers”) OR TÓPICO: (“Teenager”) OR TÓPICO: (“Youth”) OR TÓPICO: (“Youths”) OR TÓPICO: (“Female Adolescent”) OR TÓPICO: (“Female Adolescents”) OR TÓPICO: (“Male Adolescent”) OR TÓPICO: (“Male Adolescents”) OR TÓPICO: (“Boy”) OR TÓPICO: (“Boys”) OR TÓPICO: (“Girl”) OR TÓPICO: (“Girls”) OR TÓPICO: (Schools Nursery*) OR TÓPICO: (“Nursery schools”) OR TÓPICO: (“Nursery school”) OR TÓPICO: (Child Preschool*) OR TÓPICO: (“Preschool Child”) OR TÓPICO: (“Preschool Children”) OR TÓPICO: (“Day care”) OR TÓPICO: (“Kindergarten”) OR TÓPICO: (“Kindergarden”) OR TÓPICO: (“Elementary school”) OR TÓPICO: (“Schoolchild”) OR TÓPICO: (“Middle school”) OR TÓPICO: (“High school”) AND TÓPICO: (Open bite*) OR TÓPICO: (“Nonoclusion”) OR TÓPICO: (“Openbite”) OR TÓPICO: (“Apertognathia”) OR TÓPICO: (“Anterior open bite”) OR TÓPICO: (“Anterior open-bite”) OR TÓPICO: (“Open-bite”) AND TÓPICO: (Orthodontics Interceptive*) OR TÓPICO: (“Interceptive orthodontics”) OR TÓPICO: (Orthodontic Appliances Functional*) OR TÓPICO: (“Functional Orthodontic Appliance”) OR TÓPICO: (“Functional Orthodontic Appliance”) OR TÓPICO: (“Early orthodontic treatment”) OR TÓPICO: (“Early open bite treatment”) OR TÓPICO: (“Anterior open bite treatment”) OR TÓPICO: (“Orthopaedic treatment”) OR TÓPICO: (“Habit appliances”) OR TÓPICO: (“Fixed intraoral habit appliance”) OR TÓPICO: (“Spur”) OR TÓPICO: (“Spurs”) OR TÓPICO: (“Spur therapy”) OR TÓPICO: (“Spur appliance”) OR TÓPICO: (“Sharp spur”) OR TÓPICO: (“Sharp spurs”) OR TÓPICO: (“Tongue spur”) OR TÓPICO: (“Tongue spurs”) OR TÓPICO: (“Lingual spurs”) OR TÓPICO: (“Lingual spur”) OR TÓPICO: (“Palatal spur”) OR TÓPICO: (“Palatal spurs”) OR TÓPICO: (“Spike”) OR TÓPICO: (“Spikes”) OR TÓPICO: (“Lingual spike”) OR TÓPICO: (“Lingual spikes”) OR TÓPICO: (“Bonded spur”) OR TÓPICO: (“Bonded spurs”) OR TÓPICO: (“Spur bonded”) OR TÓPICO: (“Spurs bonded”) OR TÓPICO: (“Bonded lingual spur”) OR TÓPICO: (“Bonded lingual spurs”) OR TÓPICO: (“Bonded palatal spur”) OR TÓPICO: (“Bonded palatal spurs”) OR TÓPICO: (“Bondable lingual spur”) OR TÓPICO: (“Bondable lingual spurs”) OR TÓPICO: (“Bondable lingual tongue spur”) OR TÓPICO: (“Bondable lingual tongue spurs”) OR TÓPICO: (“Conventional spur”) OR TÓPICO: (“Conventional spurs”) OR TÓPICO: (“Conventional orthodontic spur”) OR TÓPICO: (“Conventional orthodontic spurs”) OR TÓPICO: (“Conventional lingual spur”) OR TÓPICO: (“Conventional lingual spurs”) OR TÓPICO: (“Banded spur”) OR TÓPICO: (“Banded spurs”)	47
Cochrane	#1 MeSH descriptor: [Infant] explode all trees 16427 #2 MeSH descriptor: [Pediatrics] explode all trees 659 #3 MeSH descriptor: [Child] explode all trees 3254 #4 MeSH descriptor: [Minors] explode all trees 8 #5 MeSH descriptor: [Adolescent] explode all trees 101695 #6 (“Pubescen”):ti,ab,kw OR (“Juvenile”):ti,ab,kw OR (“Pre-pubescen”):ti,ab,kw OR (“Boy”):ti,ab,kw OR (“Girl”):ti,ab,kw 5197 #7 MeSH descriptor: [Schools, Nursery] explode all trees 37 #8 MeSH descriptor: [Child, Preschool] explode all trees 1509 #9 MeSH descriptor: [Open Bite] explode all trees 33 #10 (“Anterior open bite”):ti,ab,kw OR (“Anterior open-bite”):ti,ab,kw OR (“Open-bite”):ti,ab,kw 41 #11 MeSH descriptor: [Orthodontics, Interceptive] explode all trees 70 #12 MeSH descriptor: [Orthodontic Appliances, Functional] explode all trees 184 #13 (“Early orthodontic treatment”):ti,ab,kw OR (“Early open bite treatment”):ti,ab,kw OR (“Anterior open bite treatment”):ti,ab,kw OR (“Habit appliances”):ti,ab,kw OR (“Fixed intraoral habit appliance”):ti,ab,kw 27 #14 (“Spurs”):ti,ab,kw OR (“Spur”):ti,ab,kw OR (“Spur therapy”):ti,ab,kw OR (“Spur appliance”):ti,ab,kw OR (“Sharp spurs”):ti,ab,kw 238 #15 (“Sharp spur”):ti,ab,kw OR (“Tongue spur”):ti,ab,kw OR (“Tongue spurs”):ti,ab,kw OR (“Lingual spurs”):ti,ab,kw OR (“Lingual spur”):ti,ab,kw 3 #16 (“Palatal spur”):ti,ab,kw OR (“Palatal spurs”):ti,ab,kw OR (“Spike”):ti,ab,kw OR (“Spikes”):ti,ab,kw OR (“Lingual spike”):ti,ab,kw 772 #17 (“Lingual spikes”):ti,ab,kw OR (“Bonded spur”):ti,ab,kw OR (“Bonded spurs”):ti,ab,kw OR (“Spur bonded”):ti,ab,kw OR (“Spurs bonded”):ti,ab,kw 4 #18 (“Bonded lingual spur”):ti,ab,kw OR (“Bonded lingual spurs”):ti,ab,kw OR (“Bonded palatal spur”):ti,ab,kw OR (“Bonded palatal spurs”):ti,ab,kw OR (“Bondable lingual spur”):ti,ab,kw 3 #19 (“Bondable lingual spurs”):ti,ab,kw OR (“Bondable lingual tongue spur”):ti,ab,kw OR (“Bondable lingual tongue spurs”):ti,ab,kw OR (“Conventional spur”):ti,ab,kw OR (“Conventional spurs”):ti,ab,kw 1 #20 (“Conventional orthodontic spur”):ti,ab,kw OR (“Conventional orthodontic spurs”):ti,ab,kw OR (“Conventional lingual spur”):ti,ab,kw OR (“Conventional lingual spurs”):ti,ab,kw OR (“Banded spur”):ti,ab,kw 1 #21 #1 OR #2 OR #3 OR #4 OR #5 OR #6 OR #7 OR #8 122097 #22 #9 OR #10 53 #23 #11 OR #12 OR #13 OR #14 OR #15 OR #16 OR #17 OR #18 OR #19 OR #20 1251 #24 #21 AND #22 AND #23 4	4
Lilacs	(tw:((Infant$) OR (Toddler) OR (Pediatrics$) OR (Pediatric) OR (Paediatric) OR (Child$) OR (Children) OR (Minors$) OR (Minor) OR (Adolescent$) OR (Adolescents) OR (Adolescence) OR (Teens) OR (Teen) OR (Teenagers) OR (Teenager) OR (Youth) OR (Youths) OR (Female Adolescent) OR (Female Adolescents) OR (Male Adolescent) OR (Male Adolescents) OR (Pubescen) OR (Juvenile) OR (Pre-pubescen) OR (Boy) OR (Boys) OR (Girl) OR (Girls) OR (Schools Nursery$) OR (Nursery schools) OR (Nursery school) OR (Child Preschool$) OR (Preschool Child) OR (Preschool Children) OR (Day care) OR (Kindergarten) OR (Kindergarden) OR (Elementary school) OR (Schoolchild) OR (Middle school) OR (High school))) AND (tw:((Open bite$) OR (Nonoclusion) OR (Openbite) OR (Apertognathia) OR (Anterior open bite) OR (Anterior open-bite) OR (Open-bite))) AND (tw:((Interceptive orthodontics$) OR (Functional Orthodontic Appliance$) OR (Functional Orthodontic Appliance) OR (Early orthodontic treatment) OR (Early open bite treatment) OR (Anterior open bite treatment) OR (Orthopaedic treatment) OR (Habit appliances) OR (Fixed intraoral habit appliance) OR (Spur) OR (Spurs) OR (Spur therapy) OR (Spur appliance) OR (Sharp spur) OR (Sharp spurs) OR (Tongue spur) OR (Tongue spurs) OR (Lingual spurs) OR (Lingual spur) OR (Palatal spur) OR (Palatal spurs) OR (Spike) OR (Spikes) OR (Lingual spike) OR (Lingual spikes) OR (Bonded spur) OR (Bonded spurs) OR (Spur bonded) OR (Spurs bonded) OR (Bonded lingual spur) OR (Bonded lingual spurs) OR (Bonded palatal spur) OR (Bonded palatal spurs) OR (Bondable lingual spur) OR (Bondable lingual spurs) OR (Bondable lingual tongue spurs) OR (Bondable lingual tongue spur) OR (Conventional spur) OR (Conventional spurs) OR (Conventional orthodontic spur) OR (Conventional orthodontic spurs) OR (Conventional lingual spur) OR (Conventional lingual spurs) OR (Banded spur) OR (Banded spurs)))	132
ClinicalTrials	Anterior Open Bite	6
OpenGrey	Anterior Open Bite	3
Google Scholar	Anterior open bite+(Child OR Adolescent)+Lingual spurs	422
